# Cell-Type-Specific Transcription of Innate Immune Regulators in response to HMPV Infection

**DOI:** 10.1155/2019/4964239

**Published:** 2019-10-09

**Authors:** Simon Loevenich, Jostein Malmo, Ann Magritt Liberg, Tatyana Sherstova, Youxian Li, Kristin Rian, Ingvild B. Johnsen, Marit W. Anthonsen

**Affiliations:** Department of Clinical and Molecular Medicine (IKOM), Norwegian University of Science and Technology (NTNU), 7491 Trondheim, Norway

## Abstract

Human metapneumovirus (HMPV) may cause severe respiratory disease. The early innate immune response to viruses like HMPV is characterized by induction of antiviral interferons (IFNs) and proinflammatory immune mediators that are essential in shaping adaptive immune responses. Although innate immune responses to HMPV have been comprehensively studied in mice and murine immune cells, there is less information on these responses in human cells, comparing different cell types infected with the same HMPV strain. The aim of this study was to characterize the HMPV-induced mRNA expression of critical innate immune mediators in human primary cells relevant for airway disease. In particular, we determined type I versus type III IFN expression in human epithelial cells and monocyte-derived macrophages (MDMs) and dendritic cells (MDDCs). In epithelial cells, HMPV induced only low levels of IFN-*β* mRNA, while a robust mRNA expression of IFN-*λ*s was found in epithelial cells, MDMs, and MDDCs. In addition, we determined induction of the interferon regulatory factors (IRFs) IRF1, IRF3, and IRF7 and critical inflammatory cytokines (IL-6, IP-10, and IL-1*β*). Interestingly, IRF1 mRNA was predominantly induced in MDMs and MDDCs. Overall, our results suggest that for HMPV infection of MDDCs, MDMs, NECs, and A549 cells (the cell types examined), cell type is a strong determinator of the ability of HMPV to induce different innate immune mediators. HMPV induces the transcription of IFN-*β* and IRF1 to higher extents in MDMs and MDDCs than in A549s and NECs, whereas the induction of type III IFN-*λ* and IRF7 is considerable in MDMs, MDDCs, and A549 epithelial cells.

## 1. Introduction

Human metapneumovirus (HMPV) is a negative single-stranded RNA virus that, like human respiratory syncytial virus (RSV), belongs to the family of *Pneumoviridae* [1, 2]. HMPV may cause severe lower respiratory tract infections in young children, and no vaccine or specific treatment for HMPV infection is available [3]. As the innate immune responses are essential for the antiviral host defense and activation of the adaptive immune system, their characterization is important. Much of the information on HMPV-induced immune responses has been obtained using mouse models or murine cells. HMPV mouse models have yielded valuable results, e.g., determining subsets of immune cells involved in immune responses and elucidating the pathogenesis of HMPV [4]. However, mice are known to have altered innate immune components and responses relative to human cells, e.g., by the expression of different subsets of pathogen recognition receptors (PRRs) and differences in cytokine/chemokine expression (e.g., absence of IL-8 in mice) thereby exhibiting altered cytokine networks [5, 6]. Thus, establishing innate immune responses to HMPV in relevant human primary cells is important to complement studies in the mouse model and to ultimately obtain increased knowledge on innate immune responses to HMPV in humans.

HMPV is sensed intracellularly by PRRs [3]. Depending on the cell type infected, several PRRs may trigger immune signaling in response to HMPV, such as the cytosolic RNA helicases melanoma differentiation-associated gene 5 (MDA5) and retinoic acid-inducible gene I (RIG-I) which belong to the RIG-I-like receptors (RLRs) [3]. These RLRs act through the mitochondrial antiviral-signaling protein (MAVS) located in the mitochondria or in the peroxisomes to stimulate the IRF3 and NF-*κ*B pathway or the IRF1, IRF3, and NF-*κ*B pathway, respectively [7–9]. This leads to induction of various innate immune mediators, proinflammatory cytokines, and interferons (IFNs). IFNs are potent antiviral cytokines that are critical in the first line of defense against viral infections and have important functions in shaping the adaptive response [10]. The antiviral effect of IFNs is mediated by their ability to induce the expression of interferon-stimulated genes (ISGs), such as antiviral effectors (e.g., ISG54 and viperin), PRRs, and interferon regulatory factors (IRFs), that can exert direct antiviral effects or amplify the antiviral response, thereby limiting viral replication [10]. Type III IFN-*λ*s are the most recently discovered group of IFNs and are critical in protection against viral infections at mucosal surfaces, but the cellular origins and functionality of type III IFNs in pathogen infections are still incompletely understood [11, 12]. Importantly, others have previously reported that HMPV can induce IFN-*λ*s in different human cells and mouse models [7, 13, 14]. However, a detailed characterization of HMPV-induced mRNA expression of type I IFNs versus type III IFNs has only been performed in the human epithelial cell line A549 [13, 14]. In addition, the highest inducible type III IFN, IFN-*λ*1, is a pseudogene in mice and variances between the type III IFN receptor in mice versus human tissues have been reported [15, 16].

The aim of this study was to characterize the mRNA expression of critical innate immune mediators in response to HMPV in human primary cells relevant for airway disease. Using a consistent HMPV stock, we determined the induction of type I IFNs (IFN-*β*), type III IFNs (IFN-*λ*1, IFN-*λ*2/3), inflammatory cytokines (IP-10, IL-6, and IL-1*β*), RLRs (MDA5, RIG-I), and the downstream transcription factors IRF1, 3, and 7. We found that HMPV induced the mRNA expression of IFN-*λ*s and IRF7 both in epithelial cells and in monocyte-derived macrophages (MDMs) and dendritic cells (MDDCs), whereas IFN-*β* and IRF1 expressions were predominantly induced in MDMs and MDDCs. Our results suggest that cell type is a strong determinant of HMPV-mediated induction of type I IFN but not type III IFN expression.

## 2. Materials and Methods

### 2.1. Amplification of Virus

The clinical isolate NL/17/00 (which, similarly to the CAN97-83 strain, represents the HMPV genetic lineage A2 [17]) was kindly provided by ViroNovative and Bernadette van den Hoogen, Erasmus MC (Rotterdam). LLC-MK2 (ATCC) monolayers were inoculated with low passage virus at low multiplicity of infection (m.o.i., 0.01) in OptiMEM containing 2% FBS, 20 *μ*g/mL gentamicin, 0.7 nM glutamine, and 50 *μ*g/mL trypsin. After 7-8 days, the virus was harvested from cells and supernatant by freeze-thawing at -80°C, followed by purification on a 20% sucrose cushion and resuspension in OptiMEM (2% FBS). The virus titer was determined using a cell-based immunoassay. Purified virus was serially diluted (log10) on monolayers of LLC-MK2 cells in 96-well flat-bottom plates. After four days, cells were washed, stained with LIGHT DIAGNOSTICS™ HMPV direct fluorescence assay (Merck Millipore) and focus-forming units determined by manual counting.

### 2.2. Viral Infection

A549 cells (ATCC), NECs, MDDCs, or MDMs were seeded in 48-well plates for qRT-PCR analysis or 8-well TC Lab-Tek Chamberslides (VWR) for confocal microscopy. Both A549 cells and NECs were seeded at the same cell concentration the day before infection. MDMs and MDDCs were seeded at the same cell concentration and differentiated for seven days prior to infection. On the day of infection, cells (with 60-70% confluence) were infected with HMPV at m.o.i. of 1 in the respective cell medium. Cells were incubated with virus for 6 h, 24 h, and 48 h for qPCR analysis or for 6 h and 24 h for confocal microscopy analysis. Morphology of the cells was regularly examined.

### 2.3. Cell Culture

Nasal epithelial cells (NECs) were isolated from nasal brushings of healthy donors (St. Olavs Hospital, Trondheim) as previously described [18]. Cells were grown in bronchial epithelial growth medium (BEGM) containing Lonza bronchial epithelial basal medium with Lonza BulletKit, 100 U/mL penicillin, 100 *μ*g/mL of each streptomycin and primocin. NECs were identified by immunostaining with anti-E-cadherin (Cell Signaling Technology) [19]. Monocytes were isolated from fresh buffy coats of healthy donors (blood bank of St. Olavs Hospital, Trondheim). In short, mononuclear cells were isolated using gradient centrifugation with Lymphoprep™ (Axis-Shield) and monocytes were further enriched by virtue of their attachment to a culture plate for 90 min. Enriched monocytes were cultivated for seven days in supplemented RPMI 1640 (10% human serum, 0.34 mM L-glutamine, and 10 *μ*g/mL gentamicin) with a 10 ng/mL macrophage colony-stimulating factor (M-CSF) for MDM differentiation or a 5 ng/mL granulocyte-macrophage colony-stimulating factor (GM-CSF) and 2.5 ng/mL interleukin-4 (IL-4) for differentiation into MDDCs. A549 cells were cultivated in supplemented RPMI 1640 (10% FBS, 0.7 mM L-glutamine, and 20 *μ*g/mL gentamicin).

### 2.4. qRT-PCR Analysis

RNA isolation, cDNA generation, and qRT-PCR analysis were performed as previously described [20]. Gene expression was calculated relative to GAPDH (*Δ*Ct) or as fold change (*ΔΔ*Ct). Fold change of gene expression (except for HMPV N-gene) was related to uninfected cells, whereas fold change in HMPV N-gene expression was related to 6 h HMPV treatment. The following primer sequences were used (5′-3′ orientation): HMPV (fwd) CATATAAGCATGCTATATTAAAAGAGTCTC, HMPV (rev) CCTATTTCTGCAGCATATTTGTAATCAG, IL-29/IFN-*λ*1 (fwd) GGGACCTGAGGCTTCTCC, IL-29/IFN-*λ*1 (rev) CCAGGACCTTCAGCGTCA, MDA5 (fwd) GGCACCATGGGAAGTGATT, MDA5 (rev) ATTTGGTAAGGCCTGAGCTG, RIG-I (fwd) AGAGCACTTGTGGACGCTTT, and RIG-I (rev) TGCAATGTCAATGCCTTCAT. All other primer sequences were previously published [20].

### 2.5. Immunofluorescence and Confocal Fluorescence Microscopy

Cells were fixed in PBS containing 4% paraformaldehyde (PFA) for 10 min on ice, followed by permeabilization with 0.1% saponin for 30 min at room temperature. Nonspecific antibody sites were blocked with PBS containing 10% FBS and 2.5% BSA for 60 min at room temperature. For staining, cells were incubated with antibodies diluted in blocking solution. Primary antibody against HMPV nucleoprotein (MAB80138, Millipore) was added overnight at 4°C, followed by secondary antibody GAM 647 (Molecular Probes) for 30 min at room temperature. Nuclear staining was achieved with DAPI (10 min). The number of cells was counted using particle analysis in the ImageJ software, based on DAPI labeling. Cells with positive staining for HMPV N-protein were counted manually. Infectivity was calculated as percentage of infected cells relative to the total number of cells. Image acquisition parameters remained constant during imaging, and threshold values were kept the same from image to image during the analysis. An overall error of 10% was estimated for this manual data analysis.

### 2.6. Statistics

Results are shown as mean of 2 or 3 independent experiments ± SD. For all primary cells, different donors were used for each independent experiment. MDMs and MDDCs were donor-matched, i.e., one donor was used to generate both MDMs and MDDCs (using different donors for each independent experiment). GraphPad Prism 5 was used for statistical analysis. Single comparisons between two groups were made by unpaired, two-tailed Student's *t*-test. A *P* value < 0.05 was considered statistically significant. For multiple comparisons, one-sided ANOVA with Dunnett's test was performed (confidence level 0.95). A *P* value < 0.05 was considered statistically significant.

## 3. Results

### 3.1. HMPV Infection Efficiency and Viral RNA Synthesis in Human Airway Epithelial and Immune Cells

**Figure 1:**
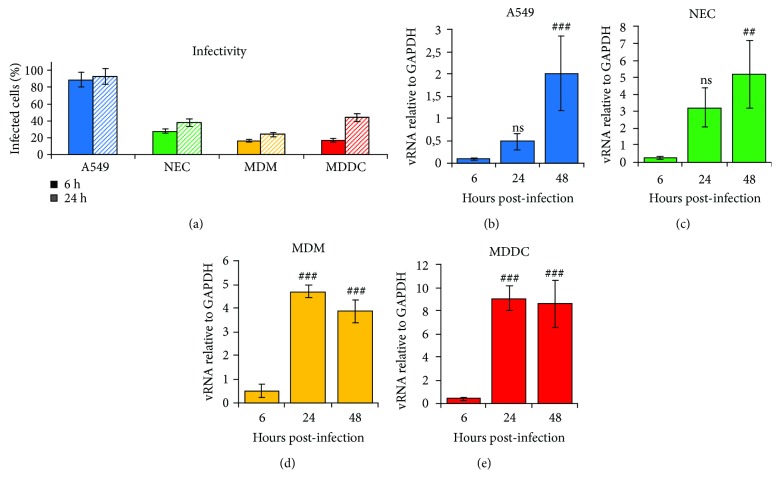
Infectivity of HMPV in airway epithelial and immune cells and kinetics of HMPV RNA accumulation. (a) Cells were infected with HMPV for 6 h or 24 h. Prior to fixation, the cells were incubated with DAPI. Intracellular staining of HMPV N-protein was performed prior to examination by confocal microscopy. Approximately, 300 cells were analyzed for each time point. Infectivity was calculated as percentage of infected cells relative to total number of cells. Experiments were performed at least two times. Error bars represent approximated error of manual data analysis. (b-e) Cells were infected for the indicated time points with HMPV. Viral N-RNA levels were analyzed by qRT-PCR. Expression was normalized relative to GAPDH. Data are presented as mean ± SD of 3 (b) or 2 (c-e) independent experiments. (b) A549, (c) NEC, (d) MDM, and (e) MDDC. Statistical analysis (one-sided ANOVA with Dunnett's test): *P* < 0.05 (#), *P* < 0.01 (##), and *P* < 0.001 (###); comparisons were made between cells at 6 h.p.i. and infected cells at 24/48 h.p.i.

To study HMPV infection and innate immune responses in human cells, we used human airway epithelial cells (A549s and NECs) and primary human immune cells (MDMs and MDDCs). The cell line A549 is frequently used in similar studies and was used herein to represent transformed alveolar epithelial cells. Cultures of primary NECs were established from nasal epithelia using a previously established protocol [18]. Human monocytes from blood donors were differentiated with M-CSF to produce MDMs or with GM-CSF and IL-4 to generate MDDCs as reported previously [21]. Initially, we characterized HMPV infectivity and viral replication in these cells. Cells were infected with HMPV NL/17/00 (A2 lineage) for 6 h or 24 h prior to intracellular staining of HMPV N-protein, confocal microscopy, and determination of infectivity. In HMPV-infected cells, we found typical intracytoplasmic granular staining for the N-protein-specific antibody (Supplementary [Supplementary-material supplementary-material-1]-[Supplementary-material supplementary-material-1]), but not when cells were stained with the isotype-specific control antibody or uninfected cells were stained for the N-protein (data not shown). Infectivity was calculated as the fraction of cells that was positive for N-protein staining. Our results show that HMPV infected primary cells with markedly lower efficiency than transformed cells (Figure 1(a)). For airway epithelial cells, about 90% of transformed A549s were infected at 24 hours postinfection (h.p.i.) whereas only about 40% of primary NECs were infected after the same infection time (Figure 1(a), Supplementary [Supplementary-material supplementary-material-1], [Supplementary-material supplementary-material-1]). Similarly, MDDCs and MDMs showed lower infectivity than A549s, with a maximum infectivity of 40% and 23% at 24 h.p.i., respectively (Figure 1(a)). Next, we evaluated the extent to which HMPV replicated in different cells by determining the level of viral RNA (vRNA) synthesis over time. Cells were infected with HMPV for 6 h, 24 h, or 48 h, and levels of intracellular vRNA were determined by qRT-PCR-mediated assessment of the HMPV N-gene. Due to the differences between infectivity of different cell types at 6 h.p.i., we determined vRNA levels relative to GAPDH and not as fold change (Figures 1(b)–1(e)). The ability of HMPV to replicate differed between the cell types. HMPV vRNA levels increased markedly between 6 and 48 h.p.i. in epithelial cells (Figures 1(b) and 1(c)) whereas HMPV replication in MDMs and MDDCs stagnated or decreased between 24 and 48 h.p.i. (Figures 1(d) and 1(e)). These results are in accordance with previous work reporting on such restricted replication of HMPV in human and mouse immune cells [22–24]. Although the viral infectivity (as determined by confocal microscopy) was lower in NECs than in A549 cells, the viral replication kinetics were comparable in both cell types (Figures 1(b) and 1(c)). This suggests that the difference in infectivity between NECs and A549 cells (observed by confocal microscopy) may be due to differences in the efficiency with which virus enters NECs and A549 cells.

### 3.2. HMPV-Mediated Induction of Type I and Type III IFNs in Human Airway Epithelial and Immune Cells

**Figure 2:**
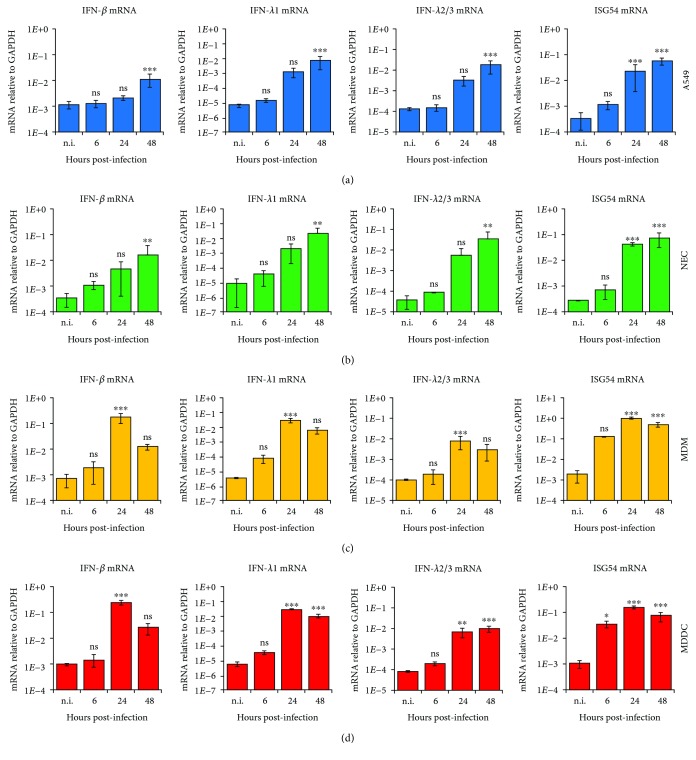
HMPV-mediated expression of the antiviral genes IFN-*β*, IFN-*λ*1, IFN-*λ*2/3, and ISG54 in airway epithelial and immune cells. Cells were infected with HMPV for the indicated time points. Gene expression was analyzed by qRT-PCR. Expression was normalized relative to GAPDH. Data are presented as mean ± SD of 3 (a) or 2 (b-d) independent experiments. (a) A549, (b) NEC, (c) MDM, and (d) MDDC. (a-d) Statistical analysis (one-sided ANOVA with Dunnett's test): *P* < 0.05 (^∗^), *P* < 0.01 (^∗∗^), and *P* < 0.001 (^∗∗∗^); comparisons were made between noninfected and infected cells.

To characterize HMPV-induced interferon responses, we determined the mRNA expression of the classical type I IFN-*β* and the more recently identified type III IFNs IFN-*λ*1 (IL-29), IFN-*λ*2 (IL-28A), and IFN-*λ*3 (IL-28B). For type I IFN responses, we chose to include IFN-*β* above IFN-*α* as IFN-*β* is produced by most cell types, such as epithelial cells and myeloid-derived cells, whereas IFN-*α* is predominantly produced by plasmacytoid dendritic cells [25]. Cells were infected with HMPV, and qRT-PCR analysis was performed 6, 24, and 48 h.p.i. Due to the high similarity between IFN-*λ*2 and IFN-*λ*3, our probes did not differentiate between the two. To account for (potential) differences in basal expression between the cell lines, mRNA expression was determined relative to GAPDH instead of fold change. As reported previously, HMPV only induced modest amounts of IFN-*β* in A549s [26] (Figure 2(a)). However, HMPV stimulated IFN-*β* efficiently in MDMs and MDDCs (Figures 2(c) and 2(d)). In contrast to IFN-*β*, IFN-*λ*1 and IFN-*λ*2/3 were potently induced by HMPV in all cell types examined (Figure 2, mid panels). Comparing the basal expression of IFN-*λ*1 and IFN-*β*, we found that the basal expression of IFN-*λ*1 was 100-fold lower than that of IFN-*β* in all cell types examined (Supplementary [Supplementary-material supplementary-material-1], [Supplementary-material supplementary-material-1]). Nevertheless, the basal expression of IFN-*λ*1 was similar in all examined cell types (Supplementary [Supplementary-material supplementary-material-1]). The basal expression of IFN-*β* mRNA did not differ between the cell types, except for NECs, which exhibited slightly lower basal expression compared to the other cell types (Supplementary [Supplementary-material supplementary-material-1]). This difference could contribute to higher fold induction of IFN-*β* mRNA in HMPV-infected NECs relative to A549 cells. Importantly, these results show that the observed differences in HMPV-induced IFN-*λ*1 and IFN-*β* mRNA expression between epithelial cells and immune cells were not due to differences in basal expression levels between the cell types. In addition to type I and III IFN mRNA expression, we determined the expression of the IFN-stimulated gene ISG54. ISG54 is not expressed or expressed at low levels in most human cells, but the gene is induced to high levels upon infection with many viruses and treatment with type I and type III IFNs, making it a useful marker of viral infections [27, 28]. ISG54 mRNA expression was robustly induced by HMPV in all cell types examined with the highest induction in MDMs and MDDCs (Figures 2(a)–2(d), right panels, supplementary [Supplementary-material supplementary-material-1]d), corresponding to the higher IFN induction in these cells.

Regarding the kinetics of induction of type I IFNs, type III IFNs, and ISG54, we found that HMPV infection resulted in distinct cell-specific kinetics (Figure 2). In A549 cells and NECs, HMPV stimulated a continuous increase of IFN-*β*, IFN-*λ*1, IFN-*λ*2/3, and ISG54 mRNAs at 6, 24, and 48 h.p.i (Figures 2(a) and 2(b)). In contrast, in MDMs and MDDCs, IFN-*β* induction decreased between 24 and 48 h.p.i., while induction of IFN-*λ*1, IFN-*λ*2/3, and ISG54 mRNA stagnated between 24 and 48 h.p.i. (Figures 2(c) and 2(d)). Based on visualization by light microscopy (indicating unchanged cellular morphology and confluence), the decrease of mRNA induction did not appear to be due to increased cell death. Furthermore, using an LDH assay, we did not observe significant differences in cell death in MDMs at 18-31 hours after HMPV infection (Supplementary [Supplementary-material supplementary-material-1]). The HMPV-stimulated expression of antiviral genes (IFN-*β*, IFN-*λ*1, IFN-*λ*2/3, and ISG54) corresponded to viral replication and was dependent on HMPV replication, as UV-inactivated HMPV did not induce the expression of IFN-*β* and IFN-*λ*1 in A549s and MDMs (Supplementary [Supplementary-material supplementary-material-1] and [Supplementary-material supplementary-material-1]).

### 3.3. Transcription of IRFs in HMPV-Infected Cells

**Figure 3:**
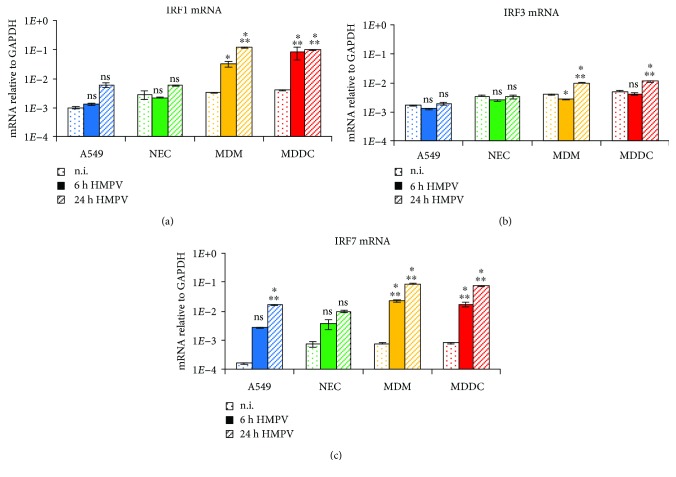
Induction of IRF1, IRF3, and IRF7 mRNA in HMPV-infected airway epithelial and immune cells. Cells were infected with HMPV for 24 h. Gene expression was analyzed by qRT-PCR. Expression was normalized relative to GAPDH. Data are presented as mean ± SD of 3 (A549 cells) or 2 (NECs, MDMs, and MDDCs) independent experiments. (a) IRF1, (b) IRF3, and (c) IRF7 mRNA. Statistical analysis (one-sided ANOVA with Dunnett's test): *P* < 0.05 (^∗^), *P* < 0.01 (^∗∗^), and *P* < 0.001 (^∗∗∗^); comparisons were made between noninfected and infected cells.

Next, we examined the effect of HMPV on the mRNA expression of IRF1, IRF3, and IRF7, a subset of IRFs that are critical for the induction of type I and type III IFNs [29]. HMPV has previously been reported to activate IRF1, IRF3, and IRF7 in A549 cells [30], and studies in mice and human MDDCs suggest that IRF7 is essential for robust induction of IFN-*α*/*β* and IFN-*λ*2/3 by HMPV [7, 14]. We found that mRNA levels of IRF3 were not substantially changed by HMPV infection in the examined cell types (Figure 3(b)). In contrast, HMPV induced the mRNA expression of IRF1 and IRF7 in a cell-type-specific manner with high induction of IRF1 mRNA in MDMs both 6 and 24 h.p.i. whereas IRF7 mRNA was substantially induced in all cells, except in NECs (Figures 3(a) and 3(c)). NECs expressed similar levels of IRF7 mRNA as A549 cells upon HMPV infection, but due to higher basal expression relative to A549 cells, these changes were not significant (Figure 3(c)).

Unlike IRF1 and IRF7, IRF3 is known to be constitutively expressed but is activated by phosphorylation at serine residue 396 in the human protein and this is decisive for IFN-*β* induction [31]. To probe HMPV-mediated activation of IRF3, we performed immunoblot analysis of p-IRF3 (Ser396). As A549 cells and NECs showed similar extent of IFN-*β* induction, while MDMs and MDDCs showed similar extent of IFN-*β* induction (which was distinct from that in A549 cells and NECs), we chose to determine IRF3 phosphorylation in A549 cells and MDMs. When comparing IRF3 Ser396-phosphorylation to total IRF3, HMPV-induced IRF3 Ser396-phosphorylation was similar in MDMs and A549 cells (Supplementary [Supplementary-material supplementary-material-1], [Supplementary-material supplementary-material-1], middle panel). However, when comparing IRF3 Ser396-phosphorylation to GAPDH, we noted that levels of phosphorylated IRF3 were considerably higher in MDMs than A549 cells even in uninfected cells (Supplementary [Supplementary-material supplementary-material-1], [Supplementary-material supplementary-material-1], right panel).

### 3.4. HMPV-Mediated Induction of Inflammatory Genes in Human Airway Epithelial and Immune Cells

**Figure 4:**
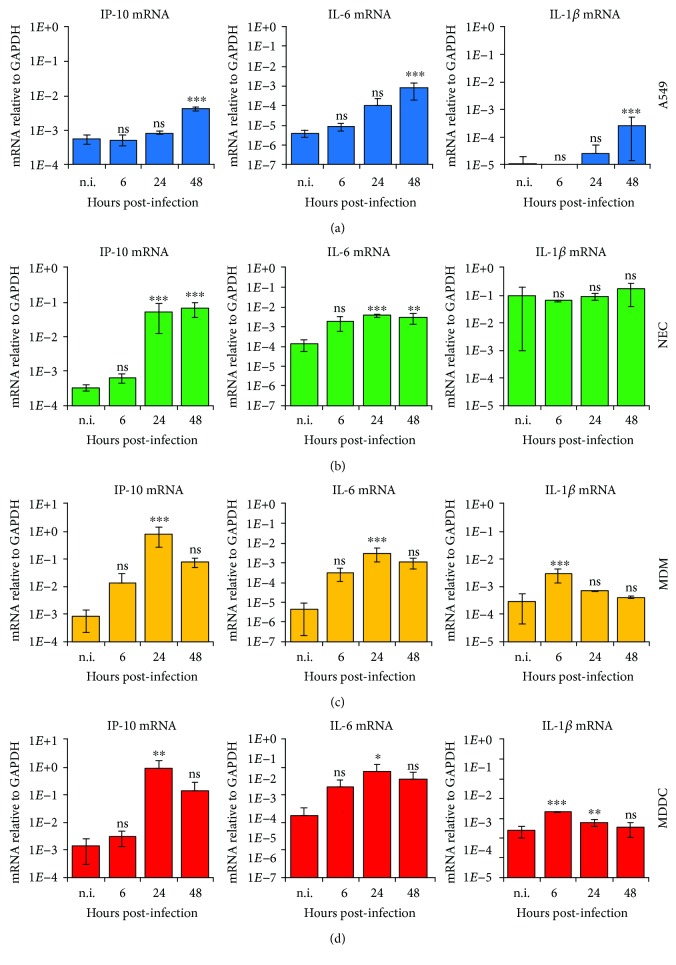
Expression of the proinflammatory genes IP-10, IL-6, and IL-1*β* in HMPV-infected airway epithelial and immune cells. Cells were infected for the indicated time points with HMPV. Gene expression was analyzed by qRT-PCR. Expression was normalized relative to GAPDH. Data are presented as mean ± SD of 3 (a) or 2 (b-d) independent experiments. (a) A549, (b) NEC, (c) MDM, and (d) MDDC. (a-d) Statistical analysis (one-sided ANOVA with Dunnett's test): *P* < 0.05 (^∗^), *P* < 0.01 (^∗∗^), and *P* < 0.001 (^∗∗∗^); comparisons were made between noninfected and infected cells.

As a readout for induction of inflammatory genes by HMPV, we determined the mRNA expression of the chemokine IFN-*γ* inducible protein 10 (IP-10), IL-6, and IL-1*β*. IP-10 and the inflammatory cytokine IL-6 contribute to the antiviral state during airway infections [23, 24, 32–34]. Interestingly, IP-10 was suggested to be a marker for susceptibility to bacterial infection in humans after administration of live-attenuated influenza vaccine [35]. We found that the basal expression of IP-10 mRNA was similar in the examined cell types, except for NECs, in which the basal IP-10 expression was slightly lower (Supplementary [Supplementary-material supplementary-material-1]e). HMPV did not induce considerable amounts of IP-10 in A549 cells (Figure 4(a)). In contrast, HMPV efficiently stimulated IP-10 mRNA in NECs, MDMs, and MDDCs at 24 h.p.i. (Figures 4(b)–4(d), left panels). Hence, A549 cells and NECs differed strongly in their ability to induce IP-10 in response to HMPV. This could potentially be explained by the increased expression of IFN-*β* in HMPV-infected NECs relative to A549 cells, as IP-10 has been reported to be induced by IFN-*β* [36]. HMPV induced the expression of IL-6 in all cell types examined and to considerably higher extent in MDMs and MDDCs (Figure 4, middle panels). HMPV-infected NECs expressed similar levels of IL-6 mRNA as MDMs, but IL-6 induction relative to uninfected cells was low in NECs (Supplementary [Supplementary-material supplementary-material-1]f). In MDMs and MDDCs, transcription of IP-10 and IL-6 mRNA decreased between 24 and 48 h.p.i. (Figures 4(c) and 4(d)), following vRNA levels at these time points. IL-1*β* mediates diverse inflammatory responses and promotes infiltration of inflammatory and immunocompetent cells from the circulation into the tissues [37]. Increased transcription of pro-IL-1*β* is the initial priming step of inflammasome/NLRP3 activation [38]. We evaluated the effect of HMPV on IL-1*β* mRNA expression in epithelial and immune cells. Overall, HMPV induced small amounts of IL-1*β* mRNA in all of the examined cell types relative to uninfected cells (Figure 4, right panels). It should be mentioned that we observed a high basal expression of IL-1*β* in NECs compared to A549 cells and MDMs/MDDCs (Supplementary [Supplementary-material supplementary-material-1]g).

### 3.5. HMPV-Mediated Induction of RLRs

**Figure 5:**
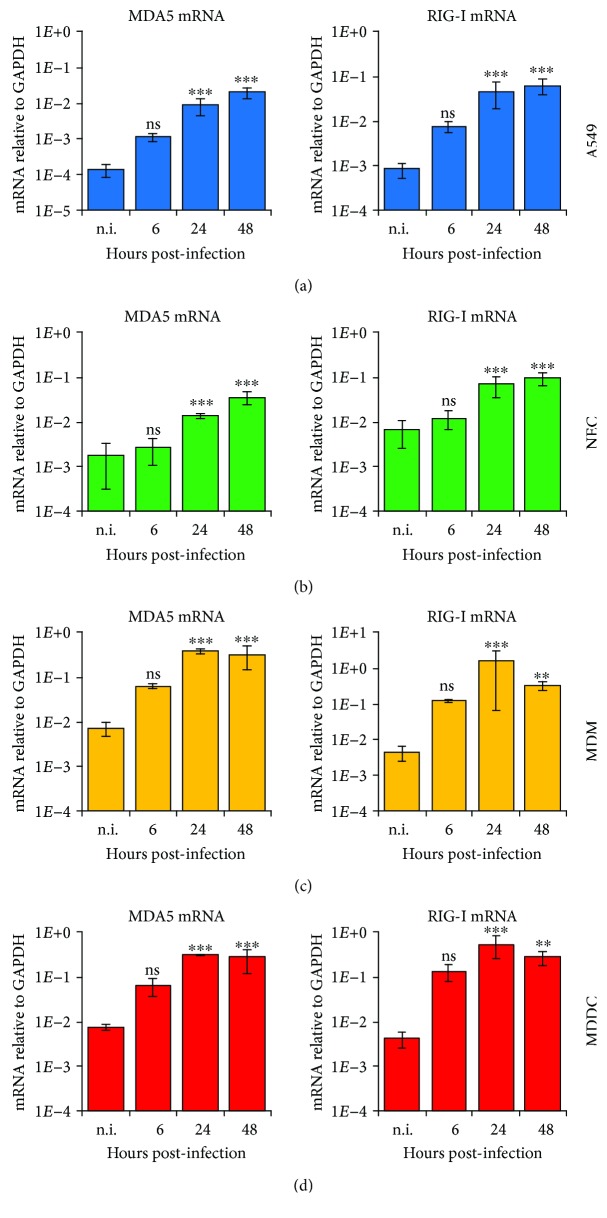
Effect of HMPV on MDA5 and RIG-I gene expression in airway epithelial and immune cells. Cells were infected with HMPV for the indicated time points. Levels of mRNA were analyzed by qRT-PCR. Cells were infected for the indicated time points with HMPV. Gene expression was analyzed by qRT-PCR. Expression was normalized relative to GAPDH. Data are presented as mean ± SD of 3 (a) or 2 (b-d) independent experiments. (a) A549, (b) NEC, (c) MDM, and (d) MDDC. (a-d) Statistical analysis (one-sided ANOVA with Dunnett's test): *P* < 0.05 (^∗^), *P* < 0.01 (^∗∗^), and *P* < 0.001 (^∗∗∗^); comparisons were made between noninfected and infected cells.

The cytosolic RNA helicases MDA5 and RIG-I and the cell surface-bound TLR4 have been reported to sense HMPV [7–9, 39, 40]. Of these, the RLRs MDA5 and RIG-I are believed to be the most relevant for HMPV-mediated signaling [7]. In addition, our preliminary data showed that HMPV did not significantly affect TLR4 mRNA expression (data not shown). Hence, we determined the induction of MDA5 and RIG-I mRNA expression by HMPV. In A549s and NECs, HMPV induced MDA5 and RIG-I mRNA to similar extent (Figures 5(a) and 5(b)). However, as the baseline expression of RIG-I in these cells was tenfold higher than that of MDA5 (Supplementary [Supplementary-material supplementary-material-1]h, [Supplementary-material supplementary-material-1]), fold induction (relative to uninfected cells) was higher for MDA5. In MDMs and MDDCs, RIG-I mRNA HMPV induced MDA5 and RIG-I mRNA to similar extent (Figures 5(c) and 5(d)). In contrast to A549 cells and NECs, the baseline expression of RIG-I in these cells was lower than that of MDA5 (Supplementary [Supplementary-material supplementary-material-1]h, [Supplementary-material supplementary-material-1]). Both RIG-I and MDA5 were induced to higher extent by HMPV in immune cells than in epithelial cells, with different kinetics compared to A549s and NECs (Figures 5(c) and 5(d)).

## 4. Discussion

The aim of this study was to characterize HMPV-induced mRNA expression of critical innate immune mediators in human primary cells relevant for airway disease. Our findings on HMPV-induced IFN expression corroborate a previous report by van den Hoogen et al. showing that HMPV has low propensity to stimulate IFN-*β* expression in airway epithelial cells [26]. However, HMPV efficiently stimulated the IFN-*β* mRNA expression in MDMs and MDDCs. Robust induction of IFN-*β* by HMPV in MDDCs has also been reported by others [7]. Thus, our results suggest that the extent of HMPV-induced IFN-*β* mRNA expression is strongly cell-type-dependent. In contrast to IFN-*β*, we found that HMPV potently induced the IFN-*λ*1 and IFN-*λ*2/3 mRNA expression both in epithelial and immune cells.

In contrast to our findings, others, e.g., Bao et al. [30] and Banos-Lara et al. (2015), observed robust induction of IFN-*β* in A549 cells by HMPV [14, 30]. In relation to Bao et al. [30], the higher IFN-*β* induction in their study compared to in our study might be due to the use of different infection protocols. Bao et al. [30] infected A549 cells with HMPV at m.o.i. 3 (compared to m.o.i. 1 in our study) using media containing trypsin [30]. Trypsin would facilitate viral infection [24], which would promote increased induction of IFN-*β*. Furthermore, Bao et al. reported robust induction of IP-10 mRNA and protein compared to our study [13, 30]. As IFN-*β* has been reported to induce IP-10 [36], it is possible that the difference in IP-10 induction between Bao et al. [30] and our study might be due to the higher IFN-*β* induction observed in their study. In relation to Banos-Lara et al. (2015), it is difficult to speculate on the reasons for differences in IFN-*β* induction as information on the experimental protocols used for viral infection (e.g., media) and virus propagation (e.g. m.o.i.) were not provided [14].

Van den Hoogen et al. showed that levels of IFNs relate to defective particles in the virus stock [26]. In order to minimize the level of defective interfering particles (DIPs) in our virus stock, we followed the procedures recommended by van den Hoogen et al. [26], i.e., using low passage virus and infecting at low multiplicity of infection (m.o.i., 0.01). Furthermore, the IFN-inducing capacity of DIPs has been shown to be unaffected by UV treatment [41–43]. We found that in A549 cells and MDMs treated with UV-inactivated HMPV, IFN-*β* and IFN-*λ*1 mRNA expressions were strongly reduced compared to cells infected with wild-type HMPV (Supplementary [Supplementary-material supplementary-material-1] b, c). Based on these two aspects, we suggest that our HMPV stock did not contain significant amounts of DIPs.

Similar to our finding that HMPV potently induces IFN-*λ* expression in both epithelial and immune cells, others have reported that influenza virus, rhinovirus, RSV, and herpes simplex virus 2 potently stimulate IFN-*λ* expression in a range of epithelial and immune cells [44–48].

Studies on influenza and RSV in epithelial cells and immune cells show a similar difference in IFN-*β* expression between these cell types as found for HMPV in our study [44, 45, 47, 49]. It remains to be investigated whether this difference in IFN-*λ* and IFN-*β* induction is caused by virus- or cell-specific signaling pathways. Overall, the signals and pathways that regulate IFN-*λ*s are currently incompletely understood. A recent report showed that the expression of IFN-*λ*1 is induced predominantly by peroxisomal MAVS and the transcription factor IRF1 (versus mitochondrial MAVS for IFN-*β*) [11]. Moreover, the type III IFN response correlated with an increased abundance of peroxisomes in epithelial cells [11]. Likewise, alveolar macrophages contain high levels of peroxisomes [50] which could contribute to the ability of macrophages to stimulate increased levels of type III IFNs.

The robust induction of IFN-*β* and IFN-*λ*s in HMPV-infected MDMs and MDDCs in our study could be correlated to the marked expression of both IRF1 and IRF7 in these cells. Studies in mice and human MDDCs suggest that IRF3 and IRF7 are essential for robust induction of IFN-*α*/*β* and IFN-*λ*2/3 by HMPV [7, 14]. We did not detect substantial induction of IRF3 mRNA expression by HMPV. However, we observed increased IRF3 Ser396-phosphorylation in HMPV-infected A549 cells and MDMs with higher levels of phosphorylated IRF3 in MDMs than in A549 cells. We propose that the overall elevated levels of IRF3 Ser396-phosphorylation in MDMs relative to A549 cells could contribute to the higher IFN-*β* induction observed in MDMs compared to A549 cells (Figures 2(c) and 2(d)). For IRF1, it was demonstrated that IRF1 is critical for Sendai virus- and dengue virus-triggered IFN-*λ* induction [11]. Thus, marked induction of IRF1 and IRF7 mRNA expression and IRF3 activation by HMPV could contribute to the robust expression of IFN-*β* and IFN-*λ* in MDMs and MDDCs.

Due to differences in the basal mRNA expression of RIG-I and MDA5, there was a distinct cell-type-dependent fold induction of RIG-I relative to MDA5 mRNA expression. We found that HMPV triggered higher fold induction of RIG-I than MDA5 mRNA expression in MDMs and MDDCs. RIG-I has been reported to be essential for the induction of type I IFN expression by RNA viruses in conventional DCs [51]. However, for HMPV, a study in human and murine MDDCs suggested that MDA5 is more important than RIG-I for the induction of type I and III IFNs [7]. In epithelial cells, we found that HMPV triggered higher fold induction of MDA5 than RIG-I mRNA expression. For measles virus, a paramyxovirus, forced expression of MDA5, but not RIG-I, in A549 cells increased IFN-*β* promoter activity [52]. In contrast, it was reported that RIG-I was essential for type I IFN expression in HMPV-infected A549 cells [8]. However, though the relative importance of MDA5 and RIG-I in HMPV-induced IFN expression is not fully elucidated, the cell-type-dependent fold induction of RIG-I relative to MDA5 mRNA expression in our study is in agreement with previous reports in A549 cells and MDDCs [7, 8]. Other pattern recognition receptors may additionally be involved in HMPV infection, e.g., TLR4 and TLR3 [40, 53], but we chose to focus on RIG-I-like receptors as these receptors have been shown to be critical for, and of similar importance for, innate immune sensing of RNA viruses in epithelial cells [51, 54], macrophages [55], and conventional DCs [51]. Also, we did not detect significant changes in TLR4 mRNA or protein levels (data not shown).

We observed that in airway epithelial cells, replication of HMPV increased progressively between 6 and 48 h.p.i. while the HMPV replication in MDMs and MDDCs was decreased or stagnated after 24 h of infection. This suggested that MDMs and MDDCs were susceptible to and permissive for initial infection by HMPV but did not allow for establishment of a “persistent” infection. A similar time-dependent decrease in viral replication, termed abortive infection, has been reported for HMPV in murine macrophages and murine/human DCs, as well as for RSV in murine macrophages [22–24]. The reason for this abortive infection could be the strong IFN induction in these cells (HMPV strongly stimulated IFN-*λ* and IFN-*β* mRNA expression in MDMs and MDDCs compared to epithelial cells). Indeed, administration of recombinant IFN-*λ*1 and IFN-*β* restricts HMPV replication [14, 56].

IFNs induce ISGs that lead to amplification of the antiviral mechanisms or interfere with the life cycle of individual viruses, thereby limiting viral replication [10]. Many ISGs are expressed in uninfected cells, but their expression is enhanced by IFNs acting through IFN-binding receptors, e.g., IFNAR (type I IFNs) and IFNLR1 (type III IFNs) [10]. In this study, we determined the HMPV-induced transcription of the ISGs RIG-I, MDA5, IRF1, IRF7, and IP-10. Of the time points examined, RIG-I and MDA5 showed maximum induction at an earlier time point in MDDCs and MDMs than in the epithelial cells studied (Figures 5(a)–5(d)). Previous studies, in colon organoids, showed that both type I and type III IFNs confer an antiviral state, but with distinct kinetics [57]. It was found that type I IFN signaling is characterized by an acute strong induction of ISGs, whereas the type III IFN-mediated ISG induction is characterized by a weaker induction of ISGs in a delayed manner compared to type I IFN [57]. Hence, we speculate that the differences in the induction profile of RIG-I and MDA-5 in MDMs and MDDCs may reflect that these ISGs are induced via IFN-*β* in MDMs and MDDCs, thus displaying a more rapid response (an earlier induction of RIG-I and MDA-5) than if induced by IFN-*λ*. Epithelial cells produced less IFN-*β* in response to HMPV than did MDMs and MDDCs (Figures 2(a)–2(d)). Moreover, IFNLR1 is predominantly expressed on epithelial cells [58]. A more rapid induction of ISGs would also lead to more rapid antiviral effects, which might cause the decline in HMPV replication in MDMs and MDDCs (Figures 1(d) and 1(e)), in contrast to what is observed in A549s and NECs (Figures 1(b) and 1(c)).

We used primary airway epithelial cells obtained by nasal brushing. Nasal epithelial cells are easier to obtain than bronchial epithelial cells that are limited by the availability of human lung tissue or invasiveness associated with obtaining the bronchial material. It has been reported that inflammatory changes in the nasal mucosa are similar to those observed at the bronchial level, e.g., with respect to chemokines and cytokines expressed [59, 60] and the upper airways regulate inflammation in the lower airways [61]. Hence, as for innate immune responses related to COPD [62], we suggest that epithelial cells from the upper airways constitute a useful and significant model system for HMPV-triggered responses. Comparing the expression of cytokines included in both our *in vitro* studies presented herein and our study on HMPV-infected children [20], we note that IP-10 expression was markedly enhanced in nasopharyngeal aspirates from HMPV-infected children as well as in HMPV-infected NECs (Figure 4(b)). In addition, IL-6 expression was not increased in nasopharyngeal aspirates of HMPV-infected children and was only moderately upregulated in HMPV-infected NECs (Figure 4(b)). The expression of IFN-*λ*2/3 was higher than IFN-*β* expression in NECs whereas similar expression levels of IFN-*λ*2/3 and IFN-*β* were found in nasopharyngeal aspirates [20]. It is possible that the different expression levels in nasopharyngeal aspirates relative to NECs are due to the presence of, e.g., macrophages in nasal samples, as reported by others [63].

## 5. Conclusions

In conclusion, we found that HMPV induced the differential expression of IFNs and IRFs in a cell-type-specific manner. HMPV induced the robust mRNA expression of IFN-*λ*s and IRF7 both in epithelial cells and MDMs and MDDCs whereas IFN-*β* and IRF1 expression was predominantly induced in MDMs and MDDCs. Thus, our results suggest that cell type is a strong determinant of HMPV-mediated induction of type I but not type III IFN expression.

## Data Availability

The data used to support the findings of this study are available from the corresponding author upon request.

## Supplementary Materials

Supplementary 1 S1: infectivity of HMPV in airway epithelial and immune cells. Cells were infected with HMPV for 6 h or 24 h. Prior to fixation, the cells were incubated with DAPI (blue). Intracellular staining of HMPV N-protein (yellow) was performed prior to examination by confocal microscopy. Confocal images of the indicated cell types 24 h.p.i. Scale bar represents 100 *μ*m. Experiments were performed at least two times. (a) A549, (b) NEC, (c) MDM, and (d) MDDC.

Supplementary 2 S2: HMPV-mediated expression of the antiviral, proinflammatory genes, and RLR genes in airway epithelial and immune cells. A549 cells, NECs, MDMs, or MDDCs were infected with HMPV for 24 h. Gene expression was analyzed by qRT-PCR. Expression was normalized relative to GAPDH. (a) IFN-*β*, (b) IFN-*λ*1, (c) IFN-*λ*2/3, (d) ISG54, (e) IP-10, (f) IL-6, (g) IL-1*β*, (h) MDA5, and (i) RIG-I. Data are presented as mean ± SD of 3 (a) or 2 (b-d) independent experiments. S3: viral replication and IFN-*β* induction by UV-inactivated HMPV. A549 cells (a) or MDMs (b) were infected for 24 h with HMPV or UV-inactivated HMPV. Gene expression was analyzed by qRT-PCR. IFN-*β* and IFN-*λ*1 expression was normalized against uninfected cells (n.i.). vRNA expression was normalized against the expression of HMPV at 6 h. Data are representative for at least two biological replicates. Error bars represent SD of three technical replicates. Statistical analysis: Student's *t*-test; *P* < 0.05 (^∗^), *P* < 0.01 (^∗∗^), and *P* < 0.001 (^∗∗∗^); comparisons were made between noninfected and infected (^∗^) or between HMPV and UV-HMPV (#). S4: LDH assay of HMPV-infected MDMs. Cells were infected with HMPV for the indicated time points. LDH activity was determined following the manufacturer's instruction. Maximum LDH activity was used as the positive control. The experiment was performed in duplicates. The data are presented as mean of duplicates ± SD and are representative for two independent experiments. S5: phosphorylation of IRF3 in HMPV-infected A549 cells and MDMs. A549 cells (a) or MDMs (b) were infected with HMPV for the indicated time points. Whole-cell lysates were subjected to SDS-PAGE and protein levels of phospho-IRF3 (Ser396) and total IRF3 determined by Western blot. Levels of phospho-IRF3 (Ser396) were normalized against levels of IRF3 and uninfected cells (middle panel) or only against GAPDH (right panel).

## References

[1] van den Hoogen B. G., de Jong J. C., Groen J. (2001). A newly discovered human pneumovirus isolated from young children with respiratory tract disease. *Nature Medicine*.

[2] Rima B., Collins P., Easton A. (2017). ICTV virus taxonomy profile: Pneumoviridae. *The Journal of General Virology*.

[3] Cespedes P. F., Palavecino C. E., Kalergis A. M., Bueno S. M. (2016). Modulation of host immunity by the human metapneumovirus. *Clinical Microbiology Reviews*.

[4] Cheemarla N. R., Guerrero-Plata A. (2015). Immune response to human metapneumovirus infection: what we have learned from the mouse model. *Pathogens*.

[5] Mestas J., Hughes C. C. W. (2004). Of mice and not men: differences between mouse and human immunology. *The Journal of Immunology*.

[6] Rehli M. (2002). Of mice and men: species variations of toll-like receptor expression. *Trends in Immunology*.

[7] Banos-Lara D. M. R., Ghosh A., Guerrero-Plata A. (2013). Critical role of MDA5 in the interferon response induced by human metapneumovirus infection in dendritic cells and *in vivo*. *Journal of Virology*.

[8] Liao S., Bao X., Liu T. (2008). Role of retinoic acid inducible gene-I in human metapneumovirus-induced cellular signalling. *The Journal of General Virology*.

[9] Goutagny N., Jiang Z., Tian J. (2010). Cell type-specific recognition of human metapneumoviruses (HMPVs) by retinoic acid-inducible gene I (RIG-I) and TLR7 and viral interference of RIG-I ligand recognition by HMPV-B1 phosphoprotein. *The Journal of Immunology*.

[10] Schneider W. M., Chevillotte M. D., Rice C. M. (2014). Interferon-stimulated genes: a complex web of host defenses. *Annual Review of Immunology*.

[11] Odendall C., Dixit E., Stavru F. (2014). Diverse intracellular pathogens activate type III interferon expression from peroxisomes. *Nature Immunology*.

[12] Wack A., Terczynska-Dyla E., Hartmann R. (2015). Guarding the frontiers: the biology of type III interferons. *Nature Immunology*.

[13] Bao X., Sinha M., Liu T. (2008). Identification of human metapneumovirus-induced gene networks in airway epithelial cells by microarray analysis. *Virology*.

[14] Del Rocío Baños-Lara M., Harvey L., Mendoza A. (2015). Impact and regulation of lambda interferon response in human metapneumovirus infection. *Journal of Virology*.

[15] Lasfar A., Lewis-Antes A., Smirnov S. V. (2006). Characterization of the mouse IFN-*λ* ligand-receptor system: IFN-*λ*s exhibit antitumor activity against B16 melanoma. *Cancer Research*.

[16] Hermant P., Demarez C., Mahlakõiv T., Staeheli P., Meuleman P., Michiels T. (2014). Human but not mouse hepatocytes respond to interferon-lambda *in vivo*. *PLoS One*.

[17] van den Hoogen B. G., Herfst S., Sprong L. (2004). Antigenic and genetic variability of human metapneumoviruses. *Emerging Infectious Diseases*.

[18] de Courcey F., Zholos A. V., Atherton-Watson H. (2012). Development of primary human nasal epithelial cell cultures for the study of cystic fibrosis pathophysiology. *American Journal of Physiology-Cell Physiology*.

[19] Stokes A. B., Kieninger E., Schögler A. (2014). Comparison of three different brushing techniques to isolate and culture primary nasal epithelial cells from human subjects. *Experimental Lung Research*.

[20] Malmo J., Moe N., Krokstad S. (2016). Cytokine profiles in human metapneumovirus infected children: identification of genes involved in the antiviral response and pathogenesis. *PLoS One*.

[21] Palucka K. A., Taquet N., Sanchez-Chapuis F., Gluckman J. C. (1998). Dendritic cells as the terminal stage of monocyte differentiation. *The Journal of Immunology*.

[22] Cespedes P. F., Gonzalez P. A., Kalergis A. M. (2013). Human metapneumovirus keeps dendritic cells from priming antigen-specific naive T cells. *Immunology*.

[23] Kolli D., Gupta M. R., Sbrana E. (2014). Alveolar macrophages contribute to the pathogenesis of human metapneumovirus infection while protecting against respiratory syncytial virus infection. *American Journal of Respiratory Cell and Molecular Biology*.

[24] Guerrero-Plata A., Casola A., Suarez G. (2006). Differential response of dendritic cells to human metapneumovirus and respiratory syncytial virus. *American Journal of Respiratory Cell and Molecular Biology*.

[25] Ivashkiv L. B., Donlin L. T. (2014). Regulation of type I interferon responses. *Nature Reviews Immunology*.

[26] van den Hoogen B. G., van Boheemen S., de Rijck J. (2014). Excessive production and extreme editing of human metapneumovirus defective interfering RNA is associated with type I IFN induction. *The Journal of General Virology*.

[27] Diamond M. S., Farzan M. (2013). The broad-spectrum antiviral functions of IFIT and IFITM proteins. *Nature Reviews Immunology*.

[28] Reich N. C. (2013). A death-promoting role for ISG54/IFIT2. *Journal of Interferon & Cytokine Research*.

[29] Odendall C., Kagan J. C. (2015). The unique regulation and functions of type III interferons in antiviral immunity. *Current Opinion in Virology*.

[30] Bao X., Liu T., Spetch L., Kolli D., Garofalo R. P., Casola A. (2007). Airway epithelial cell response to human metapneumovirus infection. *Virology*.

[31] Taniguchi T., Ogasawara K., Takaoka A., Tanaka N. (2001). IRF family of transcription factors as regulators of host defense. *Annual Review of Immunology*.

[32] Spurrell J. C. L., Wiehler S., Zaheer R. S., Sanders S. P., Proud D. (2005). Human airway epithelial cells produce IP-10 (CXCL10) in vitro and in vivo upon rhinovirus infection. *American Journal of Physiology-Lung Cellular and Molecular Physiology*.

[33] Zhu Z., Tang W., Ray A. (1996). Rhinovirus stimulation of interleukin-6 in vivo and in vitro. Evidence for nuclear factor kappa B-dependent transcriptional activation. *The Journal of Clinical Investigation*.

[34] Dienz O., Rud J. G., Eaton S. M. (2012). Essential role of IL-6 in protection against H1N1 influenza virus by promoting neutrophil survival in the lung. *Mucosal Immunology*.

[35] Jochems S. P., Marcon F., Carniel B. F. (2018). Inflammation induced by influenza virus impairs human innate immune control of pneumococcus. *Nature Immunology*.

[36] Farber J. M. (1997). Mig and IP-10: CXC chemokines that target lymphocytes. *Journal of Leukocyte Biology*.

[37] Dinarello C. A. (2009). Immunological and inflammatory functions of the interleukin-1 family. *Annual Review of Immunology*.

[38] Latz E., Xiao T. S., Stutz A. (2013). Activation and regulation of the inflammasomes. *Nature Reviews Immunology*.

[39] Velayutham T. S., Kolli D., Ivanciuc T., Garofalo R. P., Casola A. (2013). Critical role of TLR4 in human metapneumovirus mediated innate immune responses and disease pathogenesis. *PLoS One*.

[40] Kolli D., Bao X., Liu T. (2011). Human metapneumovirus glycoprotein G inhibits TLR4-dependent signaling in monocyte-derived dendritic cells. *Journal of Immunology*.

[41] Marcus P. I., Gaccione C. (1989). Interferon induction by viruses. XIX. Vesicular stomatitis virus—New Jersey: high multiplicity passages generate interferon-inducing, defective-interfering particles. *Virology*.

[42] Sekellick M. J., Marcus P. I. (1982). Interferon induction by viruses. VIII. Vesicular stomatitis virus: [±]DI-011 particles induce interferon in the absence of standard virions. *Virology*.

[43] Strahle L., Garcin D., Kolakofsky D. (2006). Sendai virus defective-interfering genomes and the activation of interferon-beta. *Virology*.

[44] Fox J. M., Crabtree J. M., Sage L. K., Tompkins S. M., Tripp R. A. (2015). Interferon lambda upregulates IDO1 expression in respiratory epithelial cells after influenza virus infection. *Journal of Interferon & Cytokine Research*.

[45] Okabayashi T., Kojima T., Masaki T. (2011). Type-III interferon, not type-I, is the predominant interferon induced by respiratory viruses in nasal epithelial cells. *Virus Research*.

[46] Ank N., West H., Bartholdy C., Eriksson K., Thomsen A. R., Paludan S. R. (2006). Lambda interferon (IFN-*λ*), a type III IFN, is induced by viruses and IFNs and displays potent antiviral activity against select virus infections in vivo. *Journal of Virology*.

[47] Coccia E. M., Severa M., Giacomini E. (2004). Viral infection and toll-like receptor agonists induce a differential expression of type I and lambda interferons in human plasmacytoid and monocyte-derived dendritic cells. *European Journal of Immunology*.

[48] Melchjorsen J., Siren J., Julkunen I., Paludan S. R., Matikainen S. (2006). Induction of cytokine expression by herpes simplex virus in human monocyte-derived macrophages and dendritic cells is dependent on virus replication and is counteracted by ICP27 targeting NF-*κ*B and IRF-3. *The Journal of General Virology*.

[49] Hillyer P., Mane V. P., Chen A. (2017). Respiratory syncytial virus infection induces a subset of types I and III interferons in human dendritic cells. *Virology*.

[50] Karnati S., Baumgart-Vogt E. (2008). Peroxisomes in mouse and human lung: their involvement in pulmonary lipid metabolism. *Histochemistry and Cell Biology*.

[51] Kato H., Sato S., Yoneyama M. (2005). Cell type-specific involvement of RIG-I in antiviral response. *Immunity*.

[52] Berghäll H., Sirén J., Sarkar D. (2006). The interferon-inducible RNA helicase, mda-5, is involved in measles virus-induced expression of antiviral cytokines. *Microbes and Infection*.

[53] Li Y., Lund C., Nervik I. (2018). Characterization of signaling pathways regulating the expression of pro-inflammatory long form thymic stromal lymphopoietin upon human metapneumovirus infection. *Scientific Reports*.

[54] Melchjorsen J., Jensen S. B., Malmgaard L. (2005). Activation of innate defense against a paramyxovirus is mediated by RIG-I and TLR7 and TLR8 in a cell-type-specific manner. *Journal of Virology*.

[55] Francisco E., Suthar M., Gale M., Rosenfeld A. B., Racaniello V. R. (2019). Cell-type specificity and functional redundancy of RIG-I-like receptors in innate immune sensing of coxsackievirus B3 and encephalomyocarditis virus. *Virology*.

[56] Scagnolari C., Trombetti S., Selvaggi C. (2011). *In vitro* sensitivity of human metapneumovirus to type I interferons. *Viral Immunology*.

[57] Pervolaraki K., Rastgou Talemi S., Albrecht D. (2018). Differential induction of interferon stimulated genes between type I and type III interferons is independent of interferon receptor abundance. *PLoS Pathogens*.

[58] Sommereyns C., Paul S., Staeheli P., Michiels T. (2008). IFN-lambda (IFN-*λ*) is expressed in a tissue-dependent fashion and primarily acts on epithelial cells in vivo. *PLoS Pathogens*.

[59] Bourdin A., Gras D., Vachier I., Chanez P. (2009). Upper airway · 1: allergic rhinitis and asthma: united disease through epithelial cells. *Thorax*.

[60] Braunstahl G. J., Overbeek S. E., KleinJan A., Prins J. B., Hoogsteden H. C., Fokkens W. J. (2001). Nasal allergen provocation induces adhesion molecule expression and tissue eosinophilia in upper and lower airways. *The Journal of Allergy and Clinical Immunology*.

[61] Scadding G. K., Kariyawasam H. H. (2009). Airways disease: just nosing around?. *Thorax*.

[62] Comer D. M., Elborn J. S., Ennis M. (2012). Comparison of nasal and bronchial epithelial cells obtained from patients with COPD. *PLoS One*.

[63] Jochems S. P., Piddock K., Rylance J. (2017). Novel analysis of immune cells from nasal microbiopsy demonstrates reliable, reproducible data for immune populations, and superior cytokine detection compared to nasal wash. *PLoS One*.

