# Supplementary material for: Cell-Type-Specific Transcription of Innate Immune Regulators in response to HMPV Infection
Source: Mediators Inflamm. 2019 Oct 9;2019:4964239. doi: 10.1155/2019/4964239 (PMC6803734; doi:10.1155/2019/4964239)

## Supplementary Materials 2-5

**S2:** HMPV-mediated expression of the antiviral, proinflammatory genes and RLR genes in airway epithelial and immune cells. A549 cells, NECs, MDMs or MDDCs were infected with HMPV for 24h. Gene expression was analyzed by qRT-PCR. Expression was normalized relative to GAPDH. (a) IFN-β, (b) IFN-λ1, (c) IFN-λ2/3, (d) ISG54, (e) IP-10, (f) IL-6, (g) IL-1β, (h) MDA5, (i) RIG-I. Data are presented as mean ± SD of 3 (a) or 2 (b-d) independent experiments.

**
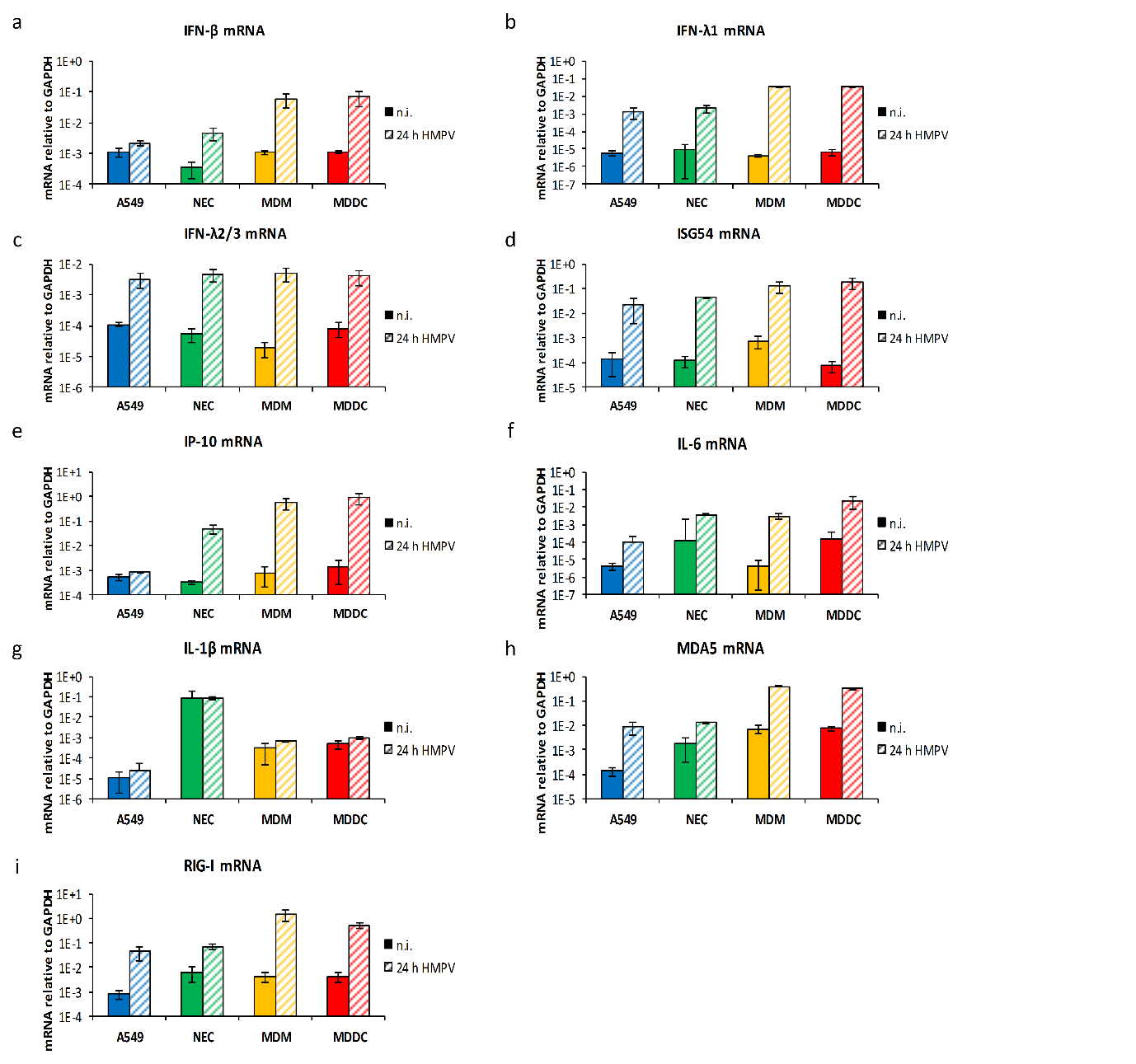
**

**S3:** Viral replication and IFN-β induction by UV-inactivated HMPV. A549 cells (a) or MDMs (b) were infected for 24h with HMPV or UV-inactivated HMPV. Gene expression was analyzed by qRT-PCR. IFN-β and IFN-λ1 expression was normalized against uninfected cells (“n.i.”). VRNA expression was normalized against expression of HMPV at 6h. Data are representative for at least two biological replicates. Error bars represent SD of three technical replicates. Statistical analysis: Student’s t-test; P < 0.05 (*), P < 0.01 (**), P < 0.001 (***); comparisons were made between non-infected and infected (*) or between HMPV and UV-HMPV (#).


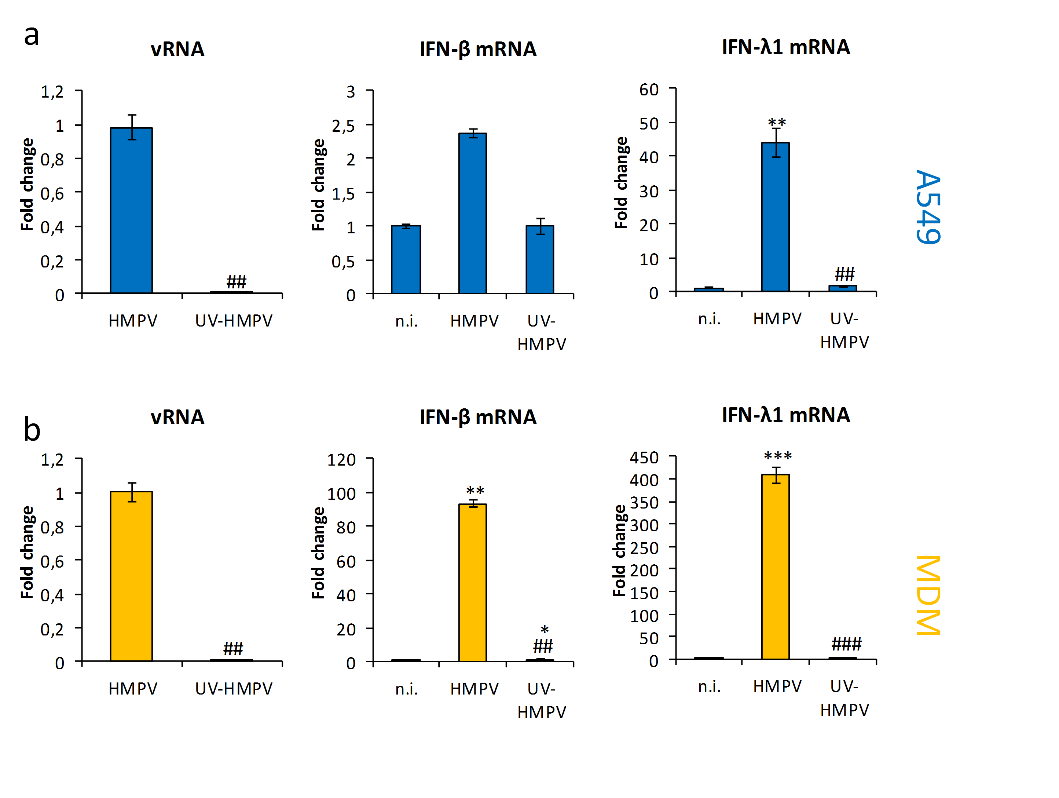


**S4:** LDH assay of HMPV-infected MDMs. Cells were infected with HMPV for the indicated timepoints. LDH activity was determined following the manufacturer’s instruction. Lysed MDMs were used as the positive control. The experiment was performed in duplicates. The data are presented as mean of duplicates ± SD and are representative for two independent experiments.


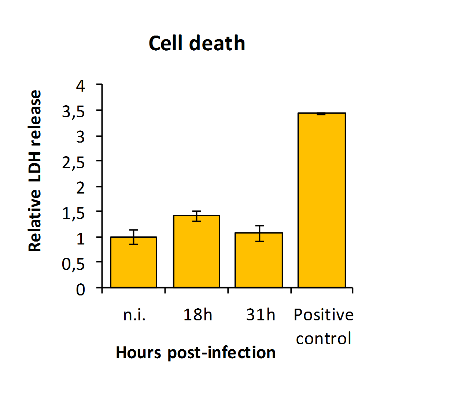


**S5:** Phosphorylation of IRF3 in HMPV-infected A549 cells and MDMs. A549 cells (a) or MDMs (b) were infected with HMPV for the indicated timepoints. Whole-cell lysates were subjected to SDS-PAGE and protein levels of phospho-IRF3(Ser396) and total IRF3 determined by Western blot. Levels of phospho-IRF3(Ser396) were normalized against levels of IRF3 and uninfected cells (middle panel) or only against GAPDH (right panel).


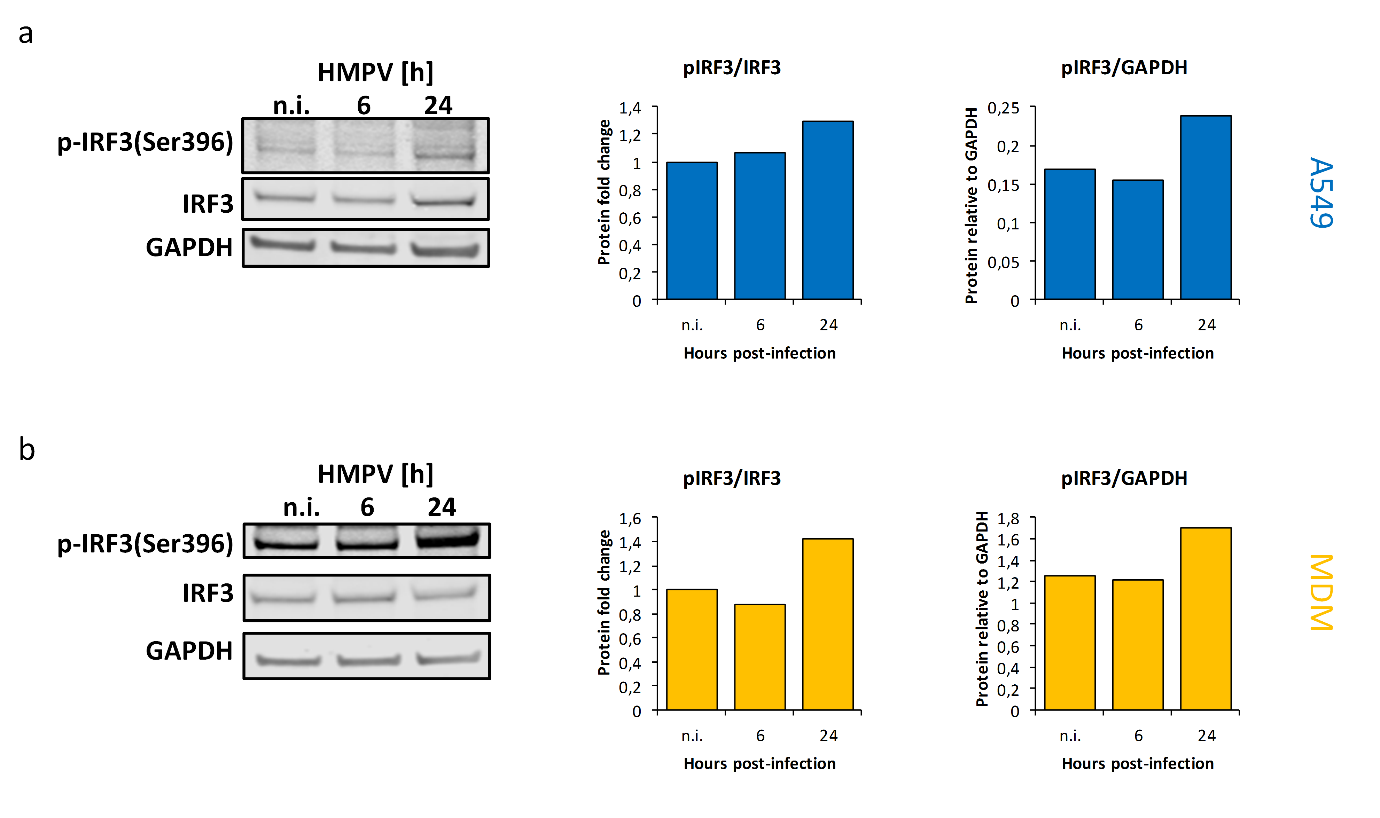

Supplement: Supplementary 2 — S2: HMPV-mediated expression of the antiviral, proinflammatory genes, and RLR genes in airway epithelial and immune cells. A549 cells, NECs, MDMs, or MDDCs were infected with HMPV for 24 h. Gene expression was analyzed by qRT-PCR. Expression was normalized relative to GAPDH. (a) IFN-β, (b) IFN-λ1, (c) IFN-λ2/3, (d) ISG54, (e) IP-10, (f) IL-6, (g) IL-1β, (h) MDA5, and (i) RIG-I. Data are presented as mean ± SD of 3 (a) or 2 (b-d) independent experiments. S3: viral replication and IFN-β induction by UV-inactivated HMPV. A549 cells (a) or MDMs (b) were infected for 24 h with HMPV or UV-inactivated HMPV. Gene expression was analyzed by qRT-PCR. IFN-β and IFN-λ1 expression was normalized against uninfected cells (n.i.). vRNA expression was normalized against the expression of HMPV at 6 h. Data are representative for at least two biological replicates. Error bars represent SD of three technical replicates. Statistical analysis: Student's t-test; P < 0.05 (∗), P < 0.01 (∗∗), and P < 0.001 (∗∗∗); comparisons were made between noninfected and infected (∗) or between HMPV and UV-HMPV (#). S4: LDH assay of HMPV-infected MDMs. Cells were infected with HMPV for the indicated time points. LDH activity was determined following the manufacturer's instruction. Maximum LDH activity was used as the positive control. The experiment was performed in duplicates. The data are presented as mean of duplicates ± SD and are representative for two independent experiments. S5: phosphorylation of IRF3 in HMPV-infected A549 cells and MDMs. A549 cells (a) or MDMs (b) were infected with HMPV for the indicated time points. Whole-cell lysates were subjected to SDS-PAGE and protein levels of phospho-IRF3 (Ser396) and total IRF3 determined by Western blot. Levels of phospho-IRF3 (Ser396) were normalized against levels of IRF3 and uninfected cells (middle panel) or only against GAPDH (right panel). [file 4964239.f2.docx]
